# Care Index, Treatment Needs, and Bilateral Occurrence of Dental Caries in the First Permanent Molars of Mexican Schoolchildren

**DOI:** 10.7759/cureus.82909

**Published:** 2025-04-24

**Authors:** Sandra I Jimenez-Gayosso, Norma L Robles-Bermeo, Rogelio J Scougall-Vilchis, Horacio Islas-Granillo, Mauricio Escoffié-Ramirez, Juan A Casanova-Sarmiento, Juan J Villalobos-Rodelo, Juan F Casanova-Rosado, Alejandro J Casanova-Rosado, Carlo E Medina-Solís

**Affiliations:** 1 Academic Area of Dentistry of Health Sciences Institute, Autonomous University of Hidalgo State, Pachuca, MEX; 2 Advanced Studies and Research Center in Dentistry "Dr. Keisaburo Miyata" School of Dentistry, Autonomous University of State of Mexico, Toluca, MEX; 3 School of Dentistry, Autonomous University of Yucatan, Yucatán, MEX; 4 School of Dentistry, Autonomous University of Campeche, Campeche, MEX; 5 School of Dentistry, Autonomous University of Sinaloa, Culiacan, MEX

**Keywords:** bilaterality, dental caries, first permanent molars, schoolchildren, treatment needs

## Abstract

Background

Dental caries is a chronic, multifactorial, and preventable disease caused by cariogenic bacteria, fermentable carbohydrates, host factors (saliva and tooth structure), and time. In Mexico, it is a public health problem, exacerbated by the lack of access to dental care. The first permanent molar (FPM), key to occlusion and mastication, is vulnerable to bilateral caries, suggesting systemic or environmental influences. Studying its bilateral pattern would help optimize preventive strategies, especially in marginalized populations with limited resources. This study aimed to determine the prevalence of caries and bilateral occurrence in the FPMs of a sample of Mexican schoolchildren, as well as to describe the Care Index and treatment needs.

Methods

A cross-sectional study was conducted. A total of 428 FPMs from 107 children (5-12 years) treated at a pediatric dental clinic of a public university in Mexico, which provides specialized services at low cost to the general population, were evaluated. The decayed, missing, and filled teeth index, the Care Index, and the Treatment Needs Index (TNI) were recorded. Symmetry tests (Stuart-Maxwell) were used to analyze bilaterality. Data were processed with Stata 14.0 (StataCorp LP, College Station, TX, USA).

Results

Of the total, 50.5% were boys, and the average age was 8.52±1.36. A total of 58.9% of children presented caries (overall decayed, missing, and filled teeth index: 1.90±2.01; in FPMs: 1.71±1.74), and 57.0% presented at least one affected FPM. It was observed that 43.0% had no caries experience in their four FPMs, and 28.0% had caries experience in all FPMs. The Care Index was 42.7%, and the TNI was 67.4%. The percentage of bilaterality was 84.1% for upper molars and 81.3% for lower molars. There were no significant differences in caries bilaterality between upper (p=0.1969) and lower (p=0.3896) molars, suggesting symmetry in the involvement.

Conclusions

Bilaterality was observed in the caries experience of the lower molars in this sample. Approximately one in four children had caries in their four FPMs. The high prevalence of caries and treatment needs underscores the urgency of affordable, preventative interventions, especially in vulnerable populations.

## Introduction

Dental caries is a preventable, chronic, non-communicable, multifactorial disease that is the most common oral health problem affecting people worldwide [1,2]. It is characterized by the interaction of cariogenic bacteria producing acid in the dental plaque biofilms, fermentable carbohydrates, and other host factors, including saliva and teeth. The organic acids produced by bacteria through the metabolism of fermentable carbohydrates lead to the demineralization of hard dental tissues. In addition to the four critical factors that directly contribute to its development, namely, cariogenic bacteria, fermentable carbohydrates, host factors (saliva and dental structure), and time, multiple factors, such as oral hygiene habits and sociodemographic factors, also influence its occurrence [1,3]. The consequences of untreated dental caries are far-reaching. If left untreated, dental caries can worsen and lead to further adverse complications such as pain, infection, tooth loss, masticatory dysfunction, and even death. Severe pain can result in difficulties eating and reduced nutritional intake, poor academic performance, behavioral issues, and hindered growth and development [1,4]. Globally, it represents a significant burden of morbidity, especially in the pediatric population. Low-income children experience disproportionately high rates of dental caries and face challenges accessing dental care as compared to their higher-income peers [5]. The high prevalence of caries worldwide has an inevitable impact on the health and well-being of children. Despite this, caries remains largely untreated due to various factors ranging from lack of knowledge to lack of access to dental care in marginalized communities [4]. In Mexico, dental caries in the pediatric population continues to be a significant public health problem, disproportionately affecting vulnerable groups [6-10].

The first permanent molar (FPM) is a fundamental pillar in the occlusion and functionality of the stomatognathic system. Its eruption around six years of age marks the beginning of permanent dentition, playing a crucial role in chewing, maxillary bone development, and guiding the eruption of posterior teeth [11,12]. Given its importance, the premature loss of the FPM or its deterioration can lead to significant consequences, such as malocclusion, facial growth disturbances, and an increased risk of caries, in adjacent teeth. Therefore, its preservation is essential to ensure comprehensive oral health in childhood and throughout life [12-15]. A poorly explored but clinically relevant aspect is the bilateral occurrence of caries in the FPMs. Bilateral occurrence suggests the possible influence of shared systemic or environmental factors, such as dietary habits, access to fluoride, or genetic predisposition, which could symmetrically increase the risk of caries [12,16,17]. Previous studies in other populations have reported patterns of bilateral occurrence in caries experience [17], reinforcing the need to investigate whether this phenomenon is replicated in Mexican schoolchildren. Understanding this pattern would not only provide valuable information about the risk of caries but could also guide more effective prevention and treatment strategies, especially in contexts where oral health resources are limited.

Despite the high prevalence of dental caries in Mexico, there is a knowledge gap concerning the specific epidemiology of FPMs in the pediatric population, particularly regarding their bilateral involvement. There are no recent data analyzing whether caries in these teeth follows a symmetric pattern or how this phenomenon relates to local or socioeconomic risk factors. Moreover, the scarcity of studies focused on treatment needs arising from this bilateral occurrence complicates the planning of effective public health policies. This study aims to contribute to closing that gap by providing evidence that allows for the design of more precise preventive and therapeutic interventions for Mexican schoolchildren. While the prevalence of caries in these teeth has been studied, information on the bilateral occurrence and its implications for treatment needs in Mexican children is limited. The study hypothesized that Mexican schoolchildren would exhibit high caries prevalence in FPMs with >50% bilateral symmetry, accompanied by elevated treatment needs and inadequate care provision.

This study aims to determine the prevalence of caries in FPMs among Mexican schoolchildren attending a public university dental clinic; analyze the bilateral occurrence patterns of these lesions; and quantify the Care Index and unmet treatment needs in this population.

## Materials and methods

Study design, population, and sample

A cross-sectional study was carried out on children between the ages of 5 and 12 years old at the Pediatric Dentistry Specialty Clinic of the Center for Research and Advanced Studies of the Autonomous University of the State of Mexico (UAEMex CIEAO). This clinic offers low-cost pediatric specialty services to the general population, with the majority of patients having a socioeconomic status that falls between low and medium. This study is a component of a project that is assessing a variety of factors related to oral health. A sample size calculation was performed using the G*Power program (version 3.1.9.7; Heinrich Heine University, Düsseldorf). The sample size calculation was performed using the following parameters: one-tailed design, medium effect size (f = 0.30), significance level of 0.05, and 90% statistical power. The methodology describing other oral health indicators from the study can be found in previous publications [18-20].

All of the following were considered for inclusion in this study: a) having at least one erupted FPM that was capable of being assessed; b) being of either sex; c) being between the ages of 5 and 12 years old; and d) being children who were living in the community, rather than being institutionalized. The following were the criteria for exclusion: (a) medical records that contained data that was unclear or information that was not obtained properly; and (b) parents who did not give their permission for clinical images to be taken. The final sample included a total of 107 patient records.

Data and variables

All data were systematically collected from medical records that had been previously completed by the patients' parents or legal guardians. The primary outcome measure - dental health status - was assessed specifically in the FPMs, as these are critical indicators of long-term oral health. Caries experience was quantified using the DMFT index (Decayed, Missing, and Filled Teeth in permanent dentition) [21], a widely accepted epidemiological tool for evaluating dental caries prevalence and treatment needs. The DMFT scores were obtained from diagnostic entries in the patients' medical histories, which were meticulously recorded by postgraduate dental students under the direct supervision of faculty members. To ensure diagnostic accuracy and minimize bias, these records were subsequently cross-referenced with high-resolution intraoral photographs taken during clinical examinations. This dual-validation approach enhanced the reliability of the data by reducing potential discrepancies in caries assessment.

The Care Index was also used for data analysis, showing the restorative care the population has been exposed to through the following relationship: CI = (filled teeth) / DMFT (100) [22]. The Treatment Needs Index (TNI) was also computed, which is obtained using the following formula: TNI = (decayed teeth) / (decayed teeth + filled teeth) (100) [23].

Statistical analysis

The statistical analysis was conducted using Stata 14.0 (StataCorp LP, College Station, TX, USA), a robust software package for data management and advanced statistical computations. In the univariate analysis, categorical variables were summarized using absolute frequencies and percentages, while continuous variables were expressed as means accompanied by their respective standard deviations (SDs) to provide a comprehensive overview of central tendency and data dispersion.

To assess the symmetry in dental health status between contralateral molars (right vs. left FPMs), the Stuart-Maxwell test and the Symmetry test were employed. These non-parametric statistical tests were selected due to their suitability for analyzing paired nominal data in square contingency tables, allowing for the detection of systematic differences in caries experience between homologous teeth. The significance level was set a priori at α = 0.05, with 95% confidence intervals reported, where applicable, to ensure rigorous interpretation of the results.

Ethical considerations

This study was conducted in strict adherence to the ethical guidelines for health research involving human participants, as established by Mexican regulatory standards and the principles outlined in the Declaration of Helsinki (2013 revision). Prior to data collection, written informed consent was obtained from all parents or legal guardians, authorizing the use of their children's anonymized clinical data for research purposes. The study protocol received formal approval from the Institutional Ethics Committee of the Faculty of Dentistry at the Autonomous University of the State of Mexico (CIEAO/16/05). Additionally, a secondary analysis of the data was reviewed and approved by the Research Ethics Committee of the Stomatology Research Network (CEIRIE-001.25), which further validated the ethical and methodological rigor of the study. All procedures were designed to safeguard participant confidentiality, with data anonymization and secure storage protocols implemented to protect personal and clinical information in accordance with current data protection regulations.

## Results

A total of 428 FPMs from 107 schoolchildren were analyzed. Of them, 50.5% were boys, and the average age was 8.52±1.36. It was observed that 43.0% had all four FPMs without caries experience, while 28.0% had caries experience in all four FPMs. The overall DMFT was 1.90±2.01, and for the FPMs, it was 1.71±1.74. The overall prevalence of caries (%DMFT > 0) was 58.9%, and for the FPMs, it was 57.0%. The Care Index was 42.7%, and the TNI was 67.4% (Table 1).

**Table 1:** Descriptive analysis of the study sample of schoolchildren SD: standard deviation; FPM: first permanent molar; DMFT: Decayed, Missing, and Filled Teeth in permanent dentition

Variables - Set 1 (Average and SD)		
Age (years)	8.52	1.36
Decayed Teeth FPMs	1.51	1.63
Missing Teeth FPMs	0.03	0.3
Filled Teeth FPMs	0.73	1.09
DMFT	1.9	2.01
DMFT-FPMs	1.71	1.74
Variables - Set 2 (n and %)		
Sex		
Boys	54	50.5
Girls	53	49.5
Number of FPMs with caries experience		
0	46	43
1	12	11.2
2	5	4.7
3	14	13.1
4	30	28
Prevalence of caries (%DMFT > 0)		
Without caries	44	41.1
With caries	63	58.9
Prevalence of caries in FPMs (%DMFT-FPMs > 0)		
Without caries	46	43
With caries	61	57
Care Index	-	42.7
Treatment Needs Index	-	67.4

Tables 2-3 show the results of the symmetry statistical test and the Stuart-Maxwell test, where both upper and lower FPMs showed statistical symmetry in health status regarding caries experience (p> 0.05, no significant difference). The percentage of bilateral occurrence was 84.1% for upper molars and 81.3% for lower FPMs.

**Table 2:** Distribution of the status of the upper FPMs in the sample n (%) Symmetry (asymptotic) p = 0.1969 Marginal homogeneity (Stuart-Maxwell) p = 0.3547 FPM: first permanent molar

	FPM left upper (26)				
FPM right upper (16)	Healthy/Sealant	Decayed	Missing	Filled	Total
Healthy/Sealant	58 (92.1)	5 (7.9)	0 (0.0)	0 (0.0)	63 (100)
Decayed	11 (28.2)	28 (71.8)	0 (0.0)	0 (0.0)	39 (100)
Missing	0 (0.0)	0 (0.0)	1 (100)	0 (0.0)	1 (100)
Filled	0 (0.0)	0 (0.0)	1 (25.0)	3 (75)	4 (100)
Total	69 (64.5)	33 (30.8)	2 (1.9)	3 (2.8)	107 (100)

**Table 3:** Distribution of the status of the lower FPMs in the sample n (%) Symmetry (asymptotic) p = 0.3896 Marginal homogeneity (Stuart-Maxwell) p = 0.2628 FPM: first permanent molar

	FPM left lower (36)				
FPM right lower (46)	Healthy/Sealant	Decayed	Missing	Filled	Total
Healthy/Sealant	49 (92.5)	4 (7.5)	0 (0.0)	0 (0.0)	53 (100)
Decayed	9 (19.1)	36 (76.6)	0 (0.0)	2 (4.3)	47 (100)
Missing	0 (0.0)	0 (0.0)	0 (0.0)	0 (0.0)	0 (100)
Filled	1 (14.3)	3 (42.8)	1 (14.3)	2 (28.6)	7 (100)
Total	59 (55.1)	43 (40.2)	1 (1.0)	4 (3.7)	107 (100)

## Discussion

This study aimed to determine the prevalence of caries and the bilateral occurrence in the FPMs, as well as to describe the Care Index and treatment needs they present. The FPM is more vulnerable to caries due to its morphological and functional characteristics, as well as the environmental conditions faced by newly erupted permanent molars. A high incidence of occlusal caries has been demonstrated in the FPM across all ages; moreover, it is the most common location for caries shortly after eruption [24]. Thus, it was found that 57.0% of participants had at least one affected FPM. No significant differences were found in the bilateral occurrence of caries between upper and lower FPMs, suggesting symmetry in the involvement. On the other hand, a low proportion of children was observed in the Care Index and a high percentage in the TNI.

Previous studies have observed that dental caries has a bilateral occurrence. For example, in children aged 12 to 13 years, the maxillary first molars (86.5%), mandibular central incisors (86.2%), and mandibular first molars (86.0%) exhibited a very high bilateral caries occurrence. In children aged 15 to 19 years, the mandibular first molars (91.6%), maxillary first molars (87.9%), and mandibular second molars (79.9%) also showed very high bilateral caries occurrence [25]. In another study, both maxillary and mandibular FPMs had a bilateral caries incidence of 80.8% and 84.0%, respectively [17]. In our study, the percentage of bilateral occurrence was similar to those previously reported by other authors [17,25]. However, the results of another study indicate that there is no statistically significant difference in the bilateral occurrence of caries in the FPM [26]. Therefore, it cannot be concluded that there is a clear tendency toward bilateral occurrence in caries of the FPM. 

Regarding the treatment needs and Care Index in the FPMs, studies have generally found high percentages of treatment needs and low percentages of the Care Index. One study evaluated the treatment needs in FPMs and found that 95.3% of children required some type of treatment [27]. Another study observed care index percentages of 23.4% and treatment needs percentages of 75.8% [28]. Previously, a study conducted in Mexico found treatment needs percentages of 92.3% and Care Index percentages of 7.1% [29]. This reflects an urgent need for an oral health program, including a component for oral health education as well as another component for dental care focused on the treatment of caries.

In this study, a low percentage of loss of FPMs was observed. However, the age of participants must be taken into account, providing the opportunity to prevent caries from progressing and leading to FPM loss since the main consequences of early extraction of FPMs include: decrease in post-extraction space; acceleration in the development and eruption of molars; caries and/or fillings in adjacent teeth; increased incidence of caries on the occlusal surfaces of adjacent teeth; effects on incisors; effects on skeletal development, such as counterclockwise rotation of the occlusal plane; slight decrease in lower anterior facial height; and a higher probability of malocclusion [15].

Based on the study findings, the importance of implementing early prevention strategies, such as pit and fissure sealants, oral health education programs, and public policies that improve access to dental services in marginalized communities, is emphasized. Given the critical role of FPMs in occlusion and facial development, their preservation is essential to avoid future complications such as malocclusion or loss of space. Future research should explore the specific determinants of bilateral caries occurrence and evaluate community interventions aimed at reducing the incidence of caries in this population.

This study has limitations that should be considered for proper interpretation. For example, participants were recruited from a low-cost university clinic, limiting the generalizability of results to other socioeconomic contexts or regions of Mexico. The inclusion of patients already seeking dental care (self-selection) could overestimate the actual prevalence of caries in the general population. Although validated with photographs, caries diagnoses depended on subjective criteria from evaluators (graduate students), without reported inter-examiner calibration. Although the statistical calculation was met, a larger sample size would allow for subgroup exploration.

## Conclusions

The results of this study demonstrate a high prevalence of caries in the first permanent molars (FPMs) of Mexican schoolchildren, with 58.9% of children affected and a Treatment Needs Index (TNI) of 67.4%. This reflects a significant gap in access to preventive and restorative dental care, particularly in populations of low socioeconomic status. The symmetry observed in the bilateral involvement of FPMs (with no statistically significant differences between sides) suggests that shared systemic or environmental factors, such as dietary habits, fluoride exposure, or genetic predisposition, could influence the development of caries similarly on both sides of the dental arch.
